# Elevated Systemic Neutrophil Count Is Associated with Diabetic Macroalbuminuria among Elderly Chinese

**DOI:** 10.1155/2015/348757

**Published:** 2015-12-30

**Authors:** Min Yang, Jun Liu, Jiong Xu, Tiange Sun, Li Sheng, Zaoping Chen, Fang Wang, Xinmei Huang, Yueyue Wu, Jianfeng Mao, Rui Zhang

**Affiliations:** Department of Endocrinology, Shanghai Fifth People's Hospital, Fudan University, Shanghai 200240, China

## Abstract

*Background.* This study investigated an association between systemic absolute neutrophil count (ANC) and albuminuria in elderly Chinese people.* Methods.* A cross-sectional study was conducted on 2265 participants attending a routine medical examination in Minhang District as part of a Platform of Chronic Disease program. Their drug history, waist circumference, height, blood pressure, fasting blood glucose, ANC, and urine albumin levels were recorded. This study conformed to the requirements of the STROBE statement.* Results.* Of the 2265 subjects, 1254 (55.4%) were diabetic and 641 (28.3%) had albuminuria. The mean ANC of patients with diabetes comorbid with macroalbuminuria was significantly higher than that of both the nondiabetic patients and patients with diabetes with lower levels of albuminuria; the latter 2 groups had statistically similar ANC. ANC significantly and positively correlated with levels of urine albumin. Based on multivariate analysis, with each 10^9^/L increase in ANC, the increase in rates of macroalbuminuria was significant but not in rates of albuminuria positivity. Based on areas under the receiver operating characteristic curve, ANC was the strongest factor predicting macroalbuminuria.* Conclusions.* Elevated ANC was associated with macroalbuminuria in diabetes, indicating that neutrophil-mediated inflammation may be involved in the exacerbation of albuminuria.

## 1. Introduction

Albuminuria (i.e., urinary albumin, or UA) is defined as an abnormally high amount of albumin in the urine and is a strong sign of early renal damage [1–4]. In type 2 diabetes and nondiabetic subjects, albuminuria is also associated with early- and long-term cardiovascular mortality [5, 6]. Elevated UA reflects a general state of widespread endothelial dysfunction and vascular damage [7–10].

Inflammation and endothelial dysfunction may be key mechanisms in the development of albuminuria [11–13]. However, evidence of an association between markers of inflammation and albuminuria is limited and controversial. In diabetic subjects with albuminuria, no differences in C-reactive protein levels (a marker of inflammation) were observed compared with healthy controls [14]. Yet in another study, serum levels of sialic acid, another marker of inflammation, correlated with increasing albumin excretion [15].

Absolute neutrophil count (ANC) is also a sensitive marker of inflammation, and the test for it is simple and inexpensive. Recently, clinical studies have shown a link between neutrophils and the development of diabetic retinopathy [16–18]. Although albuminuria is an accepted sign of early renal injury, it is not known whether the ANC is related.

We hypothesized an association between ANC and UA levels. Like the world's human population in general, China's is aging and we chose to study the elderly. Here, the UA creatinine ratio (UACR) was used as a measure of albumin excretion. Since overnight UACR correlates well with UA excretion [9], this study specifically investigated an association between systemic ANC and UACR in an elderly Chinese population.

## 2. Subjects and Methods

### 2.1. Study Participants

This community-based, cross-sectional study focused on the clinical risk factors of albuminuria. We performed the study with outpatients of our hospital in Shanghai, from March to August of 2010. All community-dwelling participants aged ≥60 years, including those residing in private residences and personal care homes, were recruited to a Platform of Chronic Disease (PCD) prevention program for a routine medical checkup. Participants who could not come to the prevention center were interviewed in their place of residence by trained personnel. Those who refused to join the study were not included in this analysis. The participants provided informed consent.

All questionnaires and procedures were approved by the Institutional Review Board in our hospital for research with human subjects. The identification of diabetes was based on the diagnostic criteria recommended by the American Diabetes Association in 1997. Potential subjects included those with a history of past diabetes or who had newly received a diagnosis by oral glucose tolerance test [19].

In total, 3053 subjects aged between 60 and 91 years (median age: 70 y) were considered for the study. The following subjects were excluded: 28 subjects presenting with painful urination, fever, cough, or skin damage; 5 subjects who were incontinent and not capable of caring for themselves; and 12 subjects with white blood cell (WBC) counts ≥10 × 10^9^/L or serum creatinine (SCr) ≥141 *μ*mol/L. Ultimately, 3003 subjects were selected for this study. Further 738 subjects had missing UA data, because they did not collect morning urine samples.

Therefore, 2265 subjects were included in the final analysis.

### 2.2. Hematological Analysis

Blood samples were collected at 8:15 a.m. after a 12-hour fast. The subjects underwent a glucose tolerance test with a 75 g oral glucose overload. Only water was permitted during the test period and no physical exercise was taken. The blood samples were collected by EDTA tube before and at 2 h after the tolerance test. After centrifugation, samples were stored at −80°C for subsequent analysis. Plasma biochemical parameters were measured in duplicate by standard enzymatic methods. Glucose, cholesterol, triglycerides, SCr, and alanine aminotransferase (ALT) levels were measured using a Dimension Vista analyzer (Siemens AG). WBC, ANC, and ANC/WBC ratio values were measured using an automated 5-category hematology analyzer (Japanese Sysmex XS1000i).

### 2.3. UA Assessment

Participants were asked to collect a random morning spot urine sample in a clean, lidded container. All urine samples were assessed centrally in a single reference laboratory at Fifth Hospital, Shanghai, China. Samples were centrifuged at 1500 ×g for 10 min and then stored at −80°C prior to analysis. Further analysis was performed with a Beckman Coulter Array 360 chemistry analyzer. Test results were considered positive for albuminuria (UA^+^) at an albumin-to-creatinine ratio (ACR) >0 mg/g; otherwise the results were accepted as negative (UA^−^; ACR = 0 mg/g). Microalbuminuria, or a moderate increase in UA, was identified as an ACR from 30 to 299 mg/g. Macroalbuminuria was defined as ACR ≥300 mg/g.

### 2.4. Physical Inspection

Blood pressure was measured twice at about 8:00 a.m., with the subject seated and with an interval of 5 min between measurements. Blood pressure measurements were taken on the right arm, which was relaxed and supported by a table, at an angle of 45° from the trunk (sphygmomanometer, Yutu, Shanghai, China). Hypertension was defined as systolic blood pressure (SBP) ≥140 mmHg or diastolic blood pressure (DBP) ≥90 mmHg.

The following measurements were recorded for all subjects: height, weight, waist circumference (horizontal position of the anterior superior iliac crest and twelfth rib edge midpoint), and hip measurement (measuring horizontally from the front of the pubic symphysis to the most convex point behind the gluteus maximus) by experienced nurses in the PCD program. Body mass index (BMI) was calculated as weight/height^2^ (kg/m^2^).

### 2.5. Statistical Analysis

Mann-Whitney *U* tests and chi-squared tests were used for analysis of continuous variables and categorical variables, respectively. The ANC levels of the following subgroups of patients were calculated and compared by analysis of variance: nondiabetic; diabetic without albuminuria; diabetic with low-level albuminuria (0 < UA < 30 mg/g); diabetic with microalbuminuria (UA 30–299 mg/g); and diabetic with macroalbuminuria (UA ≥ 300 mg/g).

The least significant difference was used for intergroup comparisons. All subjects were categorized into 5 groups according to the quintile of ANC levels (I–V). Multivariate logistic regression analysis was used to investigate an independent association between ANC and UA. Differences were considered significant at *P* ≤ 0.05.

Receiver operating characteristic (ROC) curves were plotted for factors predicting macroalbuminuria. Areas under the ROC (AUC) curve ≥0.5 were considered statistically significant (SPSS software version 17.0; SPSS, Chicago, IL, USA).

## 3. Results

The data analysis included 2265 participants aged between 60 and 91 years (median: 70 y) with a male-to-female ratio of 56.8 to 43.1 (Table 1). Compared with the 1011 participants who were free of diabetes, the 1254 participants with diabetes were older, with a higher prevalence of hypertension and increased SBP, ANC, WBC, ANC/WBC, and fasting blood glucose (FBG). The 641 participants testing positive for UACR (UA^+^) were older, with higher BMI and waist-to-hip ratio, SBP, DBP, SCr, ANC, WBC, and ANC/WBC compared with the 1624 persons testing negative for UACR (UA^−^).

**Figure 1:**
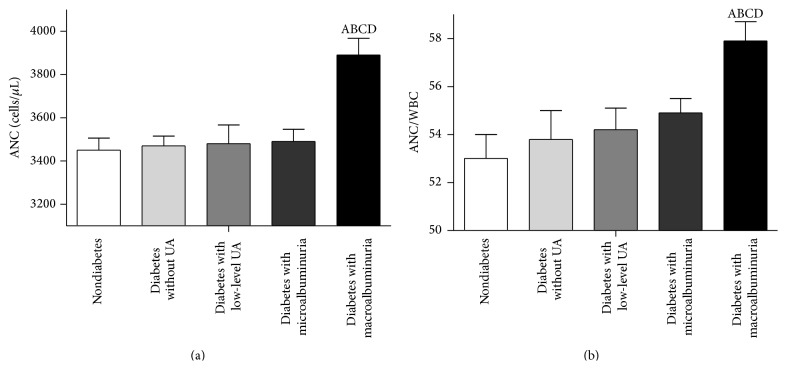
ANC and ANC/WBC ratios of groups stratified by UA severity. The ANC value and ANC/WBC ratio of the group with diabetes and macroalbuminuria were much higher than those of the other 4 groups. A: compared with nondiabetes group, *P* < 0.05. B: compared with diabetes without UA, *P* < 0.05. C: compared with diabetes with low-level UA, *P* < 0.05. D: compared with diabetes with macroalbuminuria, *P* < 0.05.

As described in Subjects and Methods, all subjects were classified as follows: nondiabetic; diabetic without albuminuria; diabetic with low-level albuminuria; diabetic with microalbuminuria; or diabetic with macroalbuminuria (Figure 1). ANC levels and ANC/WBC increased with the severity of UA (*P* < 0.0001). Patients with macroalbuminuria had significantly higher ANC and ANC/WBC counts than did the patients in the other 4 groups (*P* < 0.001). There was no statistical difference between the other 4 groups.

Regarding the results of the multivariate logistic regression analyses, after adjustments for age, BMI, gender, SBP, DBP, and SCr, the odds ratios (ORs) indicated that ANC levels significantly correlated with risk of macroalbuminuria (*P* < 0.0001). Compared with quintile I of ANC, quintile V of ANC had the highest OR value (2.311, 95% confidence interval [CI]: 1.608–3.321; Table 2).

ROC curves were plotted to evaluate the diagnostic value of age, BMI, waist-to-hip ratio, gender, SBP, DBP, ALT, SCr, total cholesterol, triglycerides, FBG, 2-hour glucose, and ANC for macroalbuminuria. ANC was the strongest predictive factor for macroalbuminuria (AUC = 0.6001) followed closely by FBG (AUC = 0.5876) and SBP (AUC = 0.5767; Table 3).

To investigate interactions among ANC, FBG, SBP, and SCr, they were grouped in separate analyses. ANC × FBG × SBP (Model 2; variables are multiplied) and ANC × FBG × SBP × SCr (Model 3) indicated 1.67% and 3.12% increase, respectively, in the predictive value for macroalbuminuria, compared with Model 1 (ANC × FBG), but these increases were not statistically significant (*P* = 0.5188 and 0.5338). Thus ANC and FBG were the most meaningful diagnostic indices for macroalbuminuria.

## 4. Discussion

This study investigated an association between systemic ANC and albuminuria in elderly Chinese people. We found that ANCs significantly increased in diabetes with macroalbuminuria, and ANC was the strongest factor predicting macroalbuminuria. These results indicate that neutrophil-mediated inflammation may be involved in the exacerbation of albuminuria.

To the best of our knowledge, this is the first report describing an association between UA and ANC levels in a population-based study. It is perplexing that the systemic neutrophil count, a very simple and inexpensive laboratory parameter, has not been reported previously in association with UA, although data from a study of 30 793 people conducted in Korea showed an association between ANC and diabetic retinopathy, after adjusting for the effects of blood pressure, blood glucose, and SCr [16].

In our present study, patients with albuminuria or those with diabetes had higher ANC levels. To study further the association between diabetes, UA, and ANC, subjects were stratified into a nondiabetic group and 4 groups with diabetes at different levels of severity of albuminuria. We found that patients with diabetes with macroalbuminuria had a notably higher mean ANC than did the other 4 groups. There were no statistical differences among the other 4 groups. In addition, ANC was an independent indicator of macroalbuminuria, but it was not an independent indicator of UA^+^. Thus, ANC might have an important role in accelerating the progression of albuminuria but not the formation of urinary protein.

Although establishing a causal mechanism for the association between ANC and albuminuria was beyond the scope of this study, the underlying link may concern vascular damage. In clinical and laboratory studies, elevated neutrophil counts were found associated with increased levels of tumor necrosis factor- (TNF-) *α*, cytokine-induced neutrophil chemotactic factor 2 alpha/beta (CINC-2*α*/*β*), interleukin- (IL-) 1*β*, IL-6, and C-reactive protein, as well as excessive production of superoxide and reactive oxygen species [20–23]. Based on the results of the present study and past investigations, we hypothesize that the systemic neutrophil count reflects the local number of neutrophils in renal vessels [24, 25]. Under the influence of proinflammatory cytokines, neutrophils in renal vessels are activated and their adhesion is intensified [26]. These responses cause abnormal leukocyte-endothelial interactions and ultimately vascular damage [27].

In the present study, compared with the other 4 quintile ANC groups, the highest level of the ANC group had the highest OR for the risk of macroalbuminuria and not quintiles II or III. This shows that the higher the ANC level, the more likely the presence of macroalbuminuria, reflecting an overtly inflammatory status in patients with macroalbuminuria [24, 27]. The exact mechanism is not clear, but whether changes in the function or shape of neutrophils are involved warrants further research.

There are several limitations to our study. First, the study design was cross-sectional and therefore we were not able to confirm a causal link between ANC and macroalbuminuria. Second, the inclusion of participants attending a routine health checkup in one community represents a potential source of selection bias, which should be considered when interpreting the results. Third, information regarding other traditional risk factors for albuminuria, such as heavy exercise [28], could not be obtained and analyzed in this study. Despite these limitations, our study involved many clinical and laboratory parameters in a large number of subjects and showed a new clinical risk factor for albuminuria in individuals, 60 years of age and older.

## 5. Conclusion

This clinical study revealed an association between elevated systemic neutrophil counts and macroalbuminuria. High levels of UA may indicate an overt inflammatory state. The potential effect of neutrophil-mediated inflammation in the progression of macroalbuminuria warrants further research.

## Figures and Tables

**Table 1:** Demographic and clinical data of patients^*∗*^.

	Diabetes			Albuminuria		
	Diabetic	Nondiabetic	*P*	UA^+^	UA^−^	*P*
Subjects, *n*	1254	1011		641	1624	
Mean age, y	71 (65–76)	70 (65–75)	0.021	70 (65–75)	70 (65–75)	0.0028
Gender, male/female	721/932	566/784	NS	266/375	717/906	NS
BMI, kg/m^2^	23.7 (21.4–26.0)	23.9 (21.6–26.3)	NS	24.0 (21.8–26.5)	23.6 (21.2–25.8)	0.0012

Waist-hip ratio	0.88 (0.84–0.91)	0.88 (0.84–0.91)	NS	0.88 (0.84–0.91)	0.87 (0.84–0.90)	0.0002
SBP, mmHg	132 (127–147)	130 (125–142)	0.0066	133 (128–148)	130 (124–140)	<0.0001
DBP, mmHg	80 (75–83)	80 (76–83)	NS	80 (76–84)	80 (74–81)	0.0019
Hypertension	774 (1254)	568 (1011)	0.0045	384 (641)	924 (1624)	NS

ACE inhibitor	73 (1254)	39 (1011)	0.0007	31 (641)	60 (1624)	NS
ARB	197 (1254)	128 (1011)	0.0218	91 (641)	222 (1624)	NS
Total cholesterol, mmol/L	5.8 (5.0–6.5)	5.8 (5.1–6.5)	NS	5.8 (5.0–6.5)	5.8 (5.1–6.5)	NS
Triglycerides, mmol/L	1.8 (1.3–2.4)	1.8 (1.3–2.4)	NS	1.8 (1.3–2.4)	1.7 (1.2–2.3)	NS

FBG, mmol/L	5.7 (5.3–7.4)	5.8 (5.5–6.2)	<0.0001	5.9 (5.4–6.6)	5.8 (5.4–6.4)	NS
Two-hour glucose, mmol/L	8.4 (6.8–12.9)	8.5 (7.9–9.3)	0.0032	8.5 (7.2–10.7)	8.4 (7.1–10.3)	NS
SCr, *µ*mol/L	69 (58–90)	70 (60–81)	NS	70 (59–2)	68.5 (60–78)	0.0038
ALT, U/L	21 (16–27)	21 (17–28)	NS	21 (16–28)	21 (16–27)	NS

WBC, 10^9^/L	6.0 (5.1–7.0)	5.9 (5.1–6.8)	0.0138	6.0 (5.1–7.0)	5.8 (5.0–6.8)	0.0131
ANC, 10^9^/L	3.5 (2.8–4.3)	3.3 (2.8–4.3)	0.0001	3.4 (2.8–4.2)	3.3 (2.7–4.0)	<0.0001
ANC/WBC, %	57.6 (50.8–63.9)	56.3 (49.8–62.3)	0.0007	58.0 (51.9–64.0)	56.2 (50.8–62.3)	0.0006
ACR, mg/g	6.4 (0–20.8)	3.2 (0–12.6)	0.0002	9.2 (27–19.2)	0	<0.0001

^*∗*^Data are presented as median and 25th–75th percentile, unless otherwise noted.

ACE, angiotensin-converting enzyme; ARB, angiotensin receptor blocker; NS, not significant.

**Table 2:** ANC quintiles in relation to the risk of macroalbuminuria and positive UA, OR (95% CI).

	ANC, 10^3^/*µ*L	Macroalbuminuria^*∗*^	UA^+^^*∗*^
I	≤2.6	Referent level	Referent level
II	2.6 to 3.2	0.996 (0.691–1.435)	1.271 (0.963–1.677)
III	3.2 to 3.7	1.458 (1.012–2.099)	1.333 (0.993–1.791)
IV	3.7 to 4.4	1.351 (0.932–1.958)	1.352 (1.000–1.827)
V	>4.4	2.311 (1.608–3.321)	1.573 (1.148–2.154)

^*∗*^ *P* for trend ≤0.0001 and 0.0653 for macroalbuminuria and UA^+^, respectively, adjusted for age, BMI, gender, SBP, DBP, and SCr.

**Table 3:** AUCs for predicting macroalbuminuria.

		AUC (95% CI)	Fold change^*∗*^	*P*
ANC, 10^3^/*µ*L		0.6001 (0.5689–0.6313)	—	—
FBG, mmol/L		0.5876 (0.5561–0.6191)	—	—
SCr, *µ*mol/L		0.5545 (0.5225–0.5869)	—	—
SBP, mmHg		0.5767 (0.5457–0.6077)	—	—

Model 1	ANC × FBG	0.6179 (0.5872–0.6486)	—	—
Model 2	ANC × FBG × SBP	0.6282 (0.6068–0.6676)	1.67	0.5188
Model 3	ANC × FBG × SBP × SCr	0.6372 (0.5978–0.6586)	3.12	0.5338

^*∗*^Compared with Model 1.

## Abbreviations

ANC: Absolute neutrophil count
ALT: Alanine aminotransferase
ACR: Albumin-to-creatinine ratio
AUC: Area under the curve
BMI: Body mass index
CINC-2*α*/*β*: Chemotactic factor 2 alpha/beta
CI: Confidence interval
DBP: Diastolic blood pressure
FBG: Fasting blood glucose
IL: Interleukin
OR: Odds ratio
PCD: Platform of Chronic Disease
ROC: Receiver operating characteristic
SCr: Serum creatinine
SBP: Systolic blood pressure
TNF: Tumor necrosis factor
UA: Urinary albumin
UACR: Urinary albumin creatinine ratio
WBC: White blood cell.
